# Robust Baseband Compression Against Congestion in Packet-Based Fronthaul Networks Using Multiple Description Coding

**DOI:** 10.3390/e21040433

**Published:** 2019-04-24

**Authors:** Seok-Hwan Park, Osvaldo Simeone, Shlomo Shamai (Shitz)

**Affiliations:** 1 Division of Electronic Engineering, Chonbuk National University, Jeonju 54896, Korea; 2 Department of Informatics, King’s College London, London WC2R2NA, UK; 3 Department of Electrical Engineering, Technion, Haifa 32000, Israel

**Keywords:** robust compression, congestion, packet-based fronthaul, multiple description coding, cloud radio access network, broadcast coding, eCPRI

## Abstract

In modern implementations of Cloud Radio Access Network (C-RAN), the fronthaul transport network will often be packet-based and it will have a multi-hop architecture built with general-purpose switches using network function virtualization (NFV) and software-defined networking (SDN). This paper studies the joint design of uplink radio and fronthaul transmission strategies for a C-RAN with a packet-based fronthaul network. To make an efficient use of multiple routes that carry fronthaul packets from remote radio heads (RRHs) to cloud, as an alternative to more conventional packet-based multi-route reception or coding, a multiple description coding (MDC) strategy is introduced that operates directly at the level of baseband signals. MDC ensures an improved quality of the signal received at the cloud in conditions of low network congestion, i.e., when more fronthaul packets are received within a tolerated deadline. The advantages of the proposed MDC approach as compared to the traditional path diversity scheme are validated via extensive numerical results.

## 1. Introduction

In a Cloud Radio Access Network (C-RAN) architecture, a cloud unit, or baseband processing unit (BBU), carries out baseband signal processing on behalf of a number of radio units, or remote radio heads (RRHs), that are connected to the cloud through an interface referred to as fronthaul links [1]. The C-RAN technology is recognized as one of the dominant architectural solutions for future wireless networks due to the promised reduction in capital and operational expenditures and the capability of large-scale interference management [2]. A major challenge of C-RAN deployment is that high-rate baseband in-phase and quadrature (IQ) samples need to be carried on the fronthaul links of limited data rate. The design of signal processing strategies, including fronthaul compression techniques, for C-RAN was widely studied in the literature [3,4,5,6].

**Figure 1:**
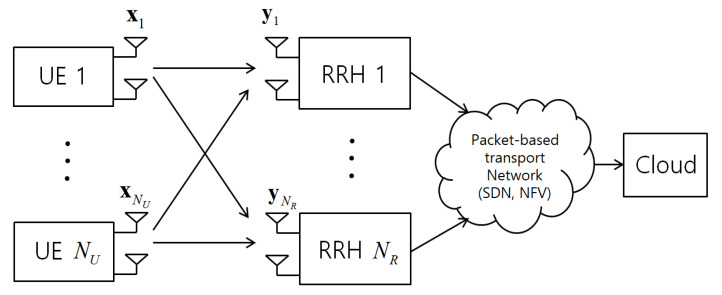
Illustration of the uplink of a Cloud Radio Access Network (C-RAN) with a packet-based fronthaul transport network.

The mentioned works [3,4,5,6] and references therein assume a conventional fronthaul topology, whereby there are dedicated point-to-point fronthaul links from the cloud to each RRH as in Common Public Radio Interface (CPRI) specification [7]. However, in modern implementations of C-RAN, as illustrated in Figure 1, the fronthaul transport network will often be packet-based and it will have a multi-hop architecture built with general-purpose switches using network function virtualization (NFV) and software-defined networking (SDN) [8,9]. Packet-based fronthaul network can leverage the wide deployment of Ethernet infrastructure [10].

Packet-based multi-hop networks are subject to congestion and packet losses. The traditional path diversity approach repeats the same packet on the multiple routes in order to mitigate these issues [11,12]. This approach can successfully reduce the packet loss probability at the cost of increasing the overhead in the fronthaul network. A limitation of these traditional schemes is that, when multiple packets arrive at the cloud within the tolerated delay, the signal quality utilized for channel decoding at the cloud is the same as if a single packet is received. To make a more efficient use of the multiple routes, in this paper, we propose a multiple description coding (MDC) scheme that operates directly on the baseband signals. Thanks to MDC, a better distortion level is obtained as more packets arrive at the cloud within the deadline. We refer to [13] for an overview and for a discussion on applications of MDC. In addition, the work [14] proposed the use of MDC to improve the achievable rate of a multicast cognitive interference channel.

Since, thanks to MDC, the signal quality varies depending on the number of packets arriving at the cloud, we propose that user equipments (UEs) leverage the broadcast approach in order to enable the adaptation of the transmission rate to the effective received signal-to-noise ratio (SNR) [15,16]. The broadcast approach defines a variable-to-fixed channel code [17] that enables the achievable rate to adapt to the channel state when the latter is known only at the receiving end. The broadcast approach splits the message of each UE into multiple submessages that are encoded independently, and transmitted as a superposition of the encoded signals. With the proposed MDC-based solution, based on the packets received within a given deadline, the cloud performs successive interference cancellation (SIC) decoding of the UEs’ submessages with a given order so that the achievable rate can be adapted to the number of delivered packets. Therefore, the number of received packets determines the quality of the channel state known only at the receiver. Related methods were introduced in [18] and [19], where broadcast coding with layered compression [20] was applied to the uplink of C-RAN systems with distributed channel state information [18] and with uncertain fronthaul capacity [19].

**Figure 2:**
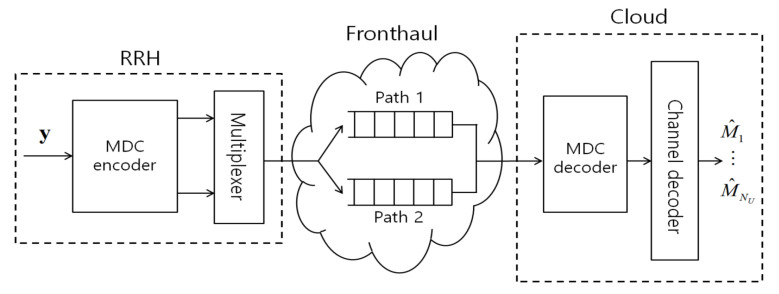
Illustration of the multiple description coding (MDC) and packet-based transport network.

More specifically, in this work, we study joint radio and fronthaul transmission for the uplink of a C-RAN with a packet-based fronthaul network. In the system, the uplink received baseband signal of each RRH is quantized and compressed producing a bit stream. The output bits are then packetized and transmitted on the fronthaul network. Following a standard approach to increase robustness to network losses and random delays (see, e.g., [11,12]), we assume that the packets are sent over multiple paths towards the cloud as seen in Figure 2. This can be done by using either conventional packet-based duplication [11,12] or the proposed MDC approach. The packets may be lost due to network delays or congestion when they are not received within a tolerable fronthaul delay dependent on the application. Based on the packets that have arrived within the delay, the cloud carries out decompression and channel decoding.

The rest of the paper is organized as follows. In Section 2, we describe the system model for the uplink of a C-RAN with packet-based fronthaul network. In Section 3, we present the proposed MDC scheme which operates in a combination with the broadcast coding. The optimization of the proposed scheme is discussed in Section 4, and the advantages of the proposed scheme are validated with extensive numerical results in Section 5. We discuss extension to general cases in Section 6, and the paper is concluded in Section 7.

We summarize some notations used throughout the paper as follows. The mutual information between random variables *X* and *Y* conditioned on *Z* is denoted as I(X;Y|Z), and h(X) denotes the differential entropy of *X*. We define CN(μ,Σ) as the circularly symmetric complex Gaussian distribution with mean μ and covariance Σ. The expectation, trace, determinant and Hermitian transpose operations are denoted by E(·), tr(·), det(·) and ${\left(\cdot \right)}^{H}$, respectively, and $C^{M\times N}$ represents the set of all M×N complex matrices. We denote as $I_{N}$ an identity matrix of size *N*, and ⊗ represents the Kronecker product. A⪰0 indicates that the matrix A is positive semidefinite.

## 2. System Model

We consider the uplink of a C-RAN in which $N_{U}$ UEs communicate with a cloud unit through $N_{R}$ RRHs. To emphasize the main idea, we first focus on the case of $N_{R}=1$ and discuss extension to a general number of RRHs in Section 6. Also, for convenience, we define the set $N_{U}≜\left\{1,\ldots ,N_{U}\right\}$ of UEs, and denote the numbers of antennas of UE *k* and of the RRH by $n_{U,k}$ and $n_{R}$, respectively. The key novel aspect as compared to the prior work reviewed above is the assumption of packet-based fronthaul connecting between RRH and cloud.

### 2.1. Uplink Wireless Channel

Each UE *k* encodes its message to be decoded at the cloud and obtains an encoded baseband signal $x_{k}∼\mathrm{CN}\left(0,\Sigma_{x_{k}}\right)\in C^{n_{U,k}\times 1}$ which is transmitted on the uplink channel toward the RRH. Assuming flat-fading channel, the signal $y\in C^{n_{R}\times 1}$ received by the RRH is given as

(1) $$y=\sum_{k\in N_{U}}H_{k}x_{k}+z=Hx+z,$$

where $H_{k}\in C^{n_{R}\times n_{U,k}}$ is the channel transfer matrix from UE *k* to the RRH, $z∼\mathrm{CN}(0,\Sigma_{z})$ is the additive noise vector, $H=[H_{1}\;\cdots \;H_{N_{U}}]$ is the channel matrix from all the UEs to the RRH, and $x={\left[x_{1}^{H}\;\cdots \;x_{N_{U}}^{H}\right]}^{H}∼\mathrm{CN}\left(0,\Sigma_{x}\right)$ is the signal transmitted by all the UEs with $\Sigma_{x}=\mathrm{diag}\left({\left\{\Sigma_{x_{k}}\right\}}_{k\in N_{U}}\right)$. We define the covariance matrix $\Sigma_{y}=H\Sigma_{x}H^{H}+\Sigma_{z}$ of y.

The RRH quantizes and compresses the received signal y producing a number of packets. As detailed next, these packets are sent to the cloud on a packet-based fronthaul network, and the cloud jointly decodes the messages sent by the UEs based on the signals received within some maximum allowed fronthaul delay.

### 2.2. Packet-Based Fronthaul Transport Network

As discussed in Section 1, in modern implementations of C-RAN, the fronthaul transport network is expected to be packet-based and to have a multi-hop architecture built with general-purpose switches using NFV and SDN [8,9]. As a result, upon compression, the received signals need to be packetized, and the packets to be transmitted on the fronthaul network to the cloud. Packets may be lost due to network delays or congestion when they are not received within a tolerated fronthaul delay dependent on the application.

**Figure 3:**
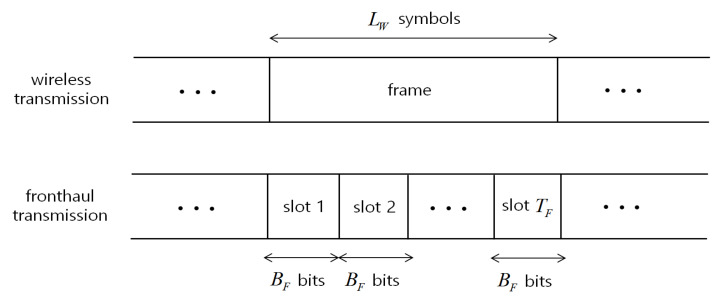
Illustration of wireless frame and fronthaul slotted transmission.

A standard approach to increase robustness to network losses and random delays is to send packets over multiple paths towards the destination (see, e.g., [11,12]). As seen in Figure 2, following [11], we model transmission on each such path, or route, as a queue. Furthermore, as seen in Figure 3, transmission on the fronthaul transport network is slotted, with each slot carrying a payload of $B_{F}$ bits. The duration of each wireless frame, of $L_{W}$ symbols, encompasses $T_{F}$ fronthaul slots. Due to congestion, each fronthaul packet sent by the RRH on route *j* takes a geometrically distributed number of time slots to be delivered. Accordingly, transmission is successful independently in each slot with probability $1-\epsilon_{F,j}$ on the *j*th route.

## 3. Robust Compression Based on Multiple Description Coding

In this section, we propose a robust compression technique based on MDC, which, in combination with broadcast coding, enables the achievable rate to be adapted to the number of packets collected by the cloud, and hence to the current network congestion level. To highlight the idea, we assume that the RRH has available two paths to the cloud. Extensions will be discussed in Section 6. The traditional path diversity approach repeats the same packet on the two routes [11]. More sophisticated forms of packet-based encoding, such as erasure coding studied in [12], are not applicable to the case of two paths. Accordingly, if one or two packets are received by the fronthaul deadline of $T_{F}$ slots, the signal is decompressed and decoding is carried out at the cloud. Note that, if both packets are received, the signal quality is the same as if one packet is received. In contrast, we propose to adopt MDC as seen in Figure 2. With MDC, if one packet is received by the deadline $T_{F}$, we obtain a certain distortion level, while we obtain a better distortion level if both packets are received ([21] Ch. 14).

In the MDC approach, the RRH first quantizes and compresses the received signal y to produce quantized signals ${\hat{y}}_{0}$, ${\hat{y}}_{1}$ and ${\hat{y}}_{2}$. Packets ${\hat{y}}_{1}$ and ${\hat{y}}_{2}$ are sent on two separate paths to the cloud, with ${\hat{y}}_{l}$ sent on route *l*. By the properties of MDC, if only a single packet l∈{1,2} arrives at the cloud within deadline $T_{F}$, the MDC decoder can recover the quantized signal ${\hat{y}}_{l}$, while the signal ${\hat{y}}_{0}$ can be recovered if the both packets are received in time.

Denote as $R_{F}$ the number of bits per symbol used to represent the signal for each of the quantized packets ${\hat{y}}_{1}$ and ${\hat{y}}_{2}$. We refer to $R_{F}$ as the compression output rate. As shown in ([21] Ch. 14), the rate $R_{F}$ should satisfy the conditions

(2) $$\begin{array}{cc} R_{F} & \geq I\left(y;{\hat{y}}_{1}\right), \end{array}$$

(3) $$\begin{array}{cc} R_{F} & \geq I\left(y;{\hat{y}}_{2}\right), \end{array}$$

(4) $$\begin{array}{cc} \mathrm{and}\;\;\;2R_{F} & \geq I\left(y;{\left\{{\hat{y}}_{l}\right\}}_{l\in \{0,1,2\}}\right)+I\left({\hat{y}}_{1};{\hat{y}}_{2}\right). \end{array}$$

To evaluate (2)–(4), as in, e.g., [3,4,5,6], we assume standard Gaussian quantization codebooks, so that the quantized signals can be modeled as

(5) $${\hat{y}}_{l}=y+q_{l},$$

for l∈{0,1,2}, where the quantization noise $q_{l}$ is independent of the signal y and distributed as $q_{l}∼\mathrm{CN}\left(0,\Omega \right)$ for l∈{1,2} and $q_{0}∼\mathrm{CN}\left(0,\Omega_{0}\right)$. The right-hand sides (RHSs) of (2)–(4) can hence be written as

(6) $$\begin{array}{cc} g_{l}\left(\Omega ,\Omega_{0}\right)= & I\left(y;{\hat{y}}_{l}\right)\\ = & \log_{2}\det\left(\Sigma_{y}+\Omega \right)-\log_{2}\det\left(\Omega \right),\;\;l\in \left\{1,2\right\}, \end{array}$$

(7) $$\begin{array}{cc} \mathrm{and}\;\;\;g_{\mathrm{sum}}\left(\Omega ,\Omega_{0}\right)= & I\left(y;{\left\{{\hat{y}}_{l}\right\}}_{l\in \{0,1,2\}}\right)+I\left({\hat{y}}_{1};{\hat{y}}_{2}\right)\\ = & h\left(y\right)+h\left({\left\{{\hat{y}}_{l}\right\}}_{l\in \{0,1,2\}}\right)-h\left(y,{\left\{{\hat{y}}_{l}\right\}}_{l\in \{0,1,2\}}\right)\\ & +h\left({\hat{y}}_{1}\right)+h\left({\hat{y}}_{2}\right)-h\left({\hat{y}}_{1},{\hat{y}}_{2}\right)\\ = & \log_{2}\det\left(\Sigma_{y}\right)+\log_{2}\det\left(A_{3}\Sigma_{y}A_{3}^{H}+\bar{\Omega }\right)\\ & -\log_{2}\det\left(A_{4}\Sigma_{y}A_{4}^{H}+\mathrm{diag}\left(0_{n_{R}},\bar{\Omega }\right)\right)\\ & +2\log_{2}\det\left(\Sigma_{y}+\Omega \right)-\log_{2}\det\left(A_{2}\Sigma_{y}A_{2}^{H}+I_{2}⊗\Omega \right), \end{array}$$

where we have defined the notations $\bar{\Omega }=\mathrm{diag}\left(\Omega_{0},I_{2}⊗\Omega \right)$ and $A_{m}=1_{m}⊗I_{n_{R}}$ with $1_{m}\in C^{m\times 1}$ denoting a column vector of all ones.

We now discuss the derivation of the probability that a packet *l* is delivered to the cloud within the given deadline $T_{F}$. The number $N_{F}$ of fronthaul packets that need to be delivered within the time $T_{F}$ to the cloud for the *l*th description is given as

(8) $$N_{F}=\left\lceil \frac{L_{W}R_{F}}{B_{F}}\right\rceil ,$$

since $L_{W}R_{F}$ is the number of bits per description and $B_{F}$ is the number of available bits per frame. Note that $N_{F}$ increases with the compression output rate $R_{F}$ and decreases with the size of the fronthaul packet $B_{F}$. Then, the probability that description l∈{1,2} sent on route *l* is received at the cloud within the deadline $T_{F}$ is given as

(9) $$P_{l}^{c}\left(T_{F}\right)=\mathrm{Pr}\left[\sum_{m=1}^{N_{F}}T_{l,m}\leq T_{F}\right],$$

where ${\left\{T_{l,m}\right\}}_{m=1}^{N_{F}}$ are independent and geometrically distributed random variables with parameter $1-\epsilon_{F,l}$ such that the sum $\sum_{m=1}^{N_{F}}T_{l,m}$ is a negative binomial random variable with parameters $1-\epsilon_{F,l}$ and $N_{F}$ ([22] Ch. 3). Therefore, the probability (9) can be written as

(10) $$P_{l}^{c}\left(T_{F}\right)=1-I_{\epsilon_{F,l}}\left(T_{F}-N_{F}+1,\;N_{F}\right),$$

where $I_{x}\left(a,b\right)$ is the regularized incomplete beta function defined as

(11) $$I_{x}\left(a,b\right)=\frac{B(x;a,b)}{B(1;a,b)},$$

with $B\left(x;a,b\right)=\int_{0}^{x}t^{a-1}{\left(1-t\right)}^{b-1}dt$. For simplicity of notation, we also define the probabilities $P_{\emptyset }^{c}\left(T_{F}\right)=\left(1-P_{1}^{c}\left(T_{F}\right)\right)\left(1-P_{2}^{c}\left(T_{F}\right)\right)$ and $P_{\mathrm{all}}^{c}\left(T_{F}\right)=P_{1}^{c}\left(T_{F}\right)P_{2}^{c}\left(T_{F}\right)$ that no or both descriptions arrive at the cloud within the deadline.

Define as M∈{0,1,2} the number of descriptions that arrive at the cloud within the given deadline $T_{F}$. The probability distribution $p_{M}\left(m\right)=\mathrm{Pr}\left[M=m\right]$ can then be written as

(12) $$p_{M}\left(m\right)=\left\{\begin{array}{cc} P_{\emptyset }^{c}\left(T_{F}\right), & m=0\\ \sum_{l=1}^{2}P_{l}^{c}\left(T_{F}\right)\left(1-P_{\bar{l}}^{c}\left(T_{F}\right)\right), & m=1\\ P_{\mathrm{all}}^{c}\left(T_{F}\right), & m=2 \end{array}\right.,$$

with the notation $\bar{1}=2$ and $\bar{2}=1$.

### Broadcast Coding

With MDC, the quality of the information available at the cloud for decoding the transmitted signals ${\left\{x_{k}\right\}}_{k\in N_{U}}$ is determined by the number *M* of descriptions that arrive at the cloud. Since the state M∈{0,1,2} is not known to the UEs, the rate cannot be a priori adapted by the UEs depending on the congestion level. To handle this issue, we propose that each UE *k* adopts a broadcast coding strategy [15,16,17,18] as

(13) $$x_{k}=x_{k,1}+x_{k,2},$$

where the signals $x_{k,1}$ and $x_{k,2}$ encode independent messages of UE *k*, and the decoder at the cloud is required to reliably recover only the signals ${\left\{x_{k,j}\right\}}_{k\in N_{U}}$ with j≤m when M=m descriptions arrive at the cloud. We denote the rate of the signal $x_{k,m}$ as $R_{k,m}$ for $k\in N_{U}$ and m∈{1,2}. We make the standard assumption that the *j*th signal $x_{k,j}$ of each UE *k* is distributed as $x_{k,j}∼\mathrm{CN}\left(0,P_{k,j}I_{n_{U,k}}\right)$, where the powers $P_{k,j}$ need to satisfy the power constraint $P_{k,1}+P_{k,2}=P$. Under the described assumption, the covariance matrix $\Sigma_{x}$ of all the transmitted signals x is given as $\Sigma_{x}=PI_{n_{U}}$ with $n_{U}=\sum_{k\in N_{U}}n_{U,k}$.

The signal $r_{m}$ collected at the cloud when M=m descriptions have arrived at the cloud is given as

(14) $$\begin{array}{cc} r_{m}= & \left\{\begin{array}{cc} 0, & m=0\\ {\hat{y}}_{1}, & m=1\\ {\hat{y}}_{0}, & m=2 \end{array}\right.. \end{array}$$

For the case of m=1, the cloud receives $r_{1}={\hat{y}}_{1}$ or $r_{1}={\hat{y}}_{2}$. In (14), we set $r_{1}={\hat{y}}_{1}$ without loss of generality, since ${\hat{y}}_{1}$ and ${\hat{y}}_{2}$ are statistically equivalent.

When no description arrives at the cloud (i.e., M=0), the cloud has no information received from the RRH, and none of the signals ${\left\{x_{k,0},x_{k,1}\right\}}_{k\in N_{U}}$ can be decoded by the cloud. When only a single description arrives at the cloud (M=1), the cloud jointly decodes the first-layer signals ${\left\{x_{k,1}\right\}}_{k\in N_{U}}$ based on the received quantized signal $r_{1}$. Therefore, the achievable sum-rate $R_{\Sigma ,1}=\sum_{k\in N_{U}}R_{k,1}$ of the first-layer signals is given as

(15) $$\begin{array}{cc} R_{\Sigma ,1} & =f_{1}\left(P,\Omega ,\Omega_{0}\right)=I\left({\bar{x}}_{1};r_{1}\right)\\ & =\log_{2}\det\left(H\Sigma_{x}H^{H}+\Sigma_{z}+\Omega \right)-\log_{2}\det\left(H{\bar{P}}_{2}H^{H}+\Sigma_{z}+\Omega \right), \end{array}$$

where we have defined the vector ${\bar{x}}_{m}={\left[x_{1,m}^{H}\;\cdots \;x_{N_{U},m}^{H}\right]}^{H}∼\mathrm{CN}\left(0,{\bar{P}}_{m}\right)$ that stacks the layer-*m* signals of all the UEs, and the notations $P={\left\{P_{k,j}\right\}}_{k\in N_{U},j\in \left\{1,2\right\}}$ and ${\bar{P}}_{m}=\mathrm{diag}\left({\left\{P_{k,m}\right\}}_{k\in N_{U}}\right)$.

If both descriptions arrive at the cloud (i.e., M=2), the cloud first jointly decodes the first-layer signals ${\left\{x_{k,1}\right\}}_{k\in N_{U}}$ from the recovered quantized signal $r_{2}$, and cancels the impact of the decoded signals from $r_{2}$, i.e., ${\tilde{r}}_{2}\leftarrow r_{2}-\sum_{k\in N_{U}}H_{k}x_{k,1}$. Then, the cloud decodes the second-layer signals ${\left\{x_{k,2}\right\}}_{k\in N_{U}}$ based on ${\tilde{r}}_{2}$. Thus, the achievable sum-rate $R_{\Sigma ,2}=\sum_{k\in N_{U}}R_{k,2}$ of the second-layer signals is given as

(16) $$\begin{array}{cc} R_{\Sigma ,2} & =f_{2}\left(P,\Omega ,\Omega_{0}\right)=I\left({\bar{x}}_{2};r_{2}|{\bar{x}}_{1}\right)\\ & =\log_{2}\det\left(H{\bar{P}}_{2}H^{H}+\Sigma_{z}+\Omega_{0}\right)-\log_{2}\det\left(\Sigma_{z}+\Omega_{0}\right). \end{array}$$

**Figure 4:**
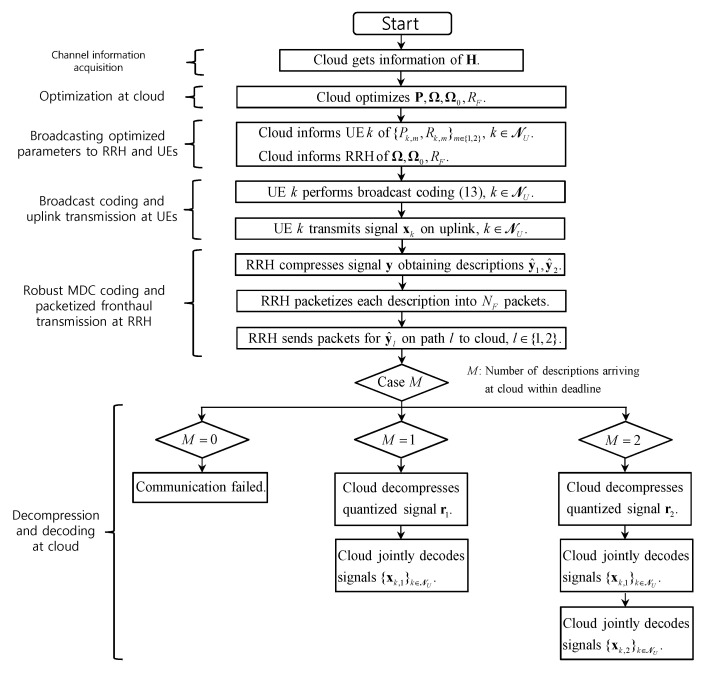
A flowchart that illustrates the operations of the proposed uplink system based on broadcast coding and multiple description coding (MDC).

In summary, the whole system operates as follows. The cloud first obtains the channel state information and optimizes the variables related to broadcast coding and MDC coding. The optimization will be discussed in Section 4. After the optimization algorithm is finished, the cloud informs the UEs and the RRH of the optimized variables. The UEs perform broadcast coding and uplink transmission, and the RRH compresses the received signal obtaining two descriptions which are packetized and sent on fronthaul paths to the cloud. Based on the received packets, the cloud performs MDC decoding of the quantized signals and SIC decoding of the UEs’ messages. We provide a flowchart that illustrates the described operations of the proposed system in Figure 4.

## 4. Problem Definition and Optimization

For fixed instantaneous channel states ${\left\{H_{k}\right\}}_{k\in N_{U}}$, we aim at jointly optimizing the compression output rate $R_{F}$, the power allocation variables P and the quantization noise covariance matrices $\left\{\Omega ,\Omega_{0}\right\}$ with the goal of maximizing the expected sum-rate denoted as ${\bar{R}}_{\Sigma }$. Here the expectation is taken with respect to the random variables ${\left\{T_{l,m}\right\}}_{l\in \left\{1,2\right\},m\in N_{F}}$ with $N_{F}=\left\{1,2,\ldots ,N_{F}\right\}$, which depend on the current congestion level of the packet network. The expected sum-rate ${\bar{R}}_{\Sigma }$ is hence given as

(17) $$\begin{array}{cc} {\bar{R}}_{\Sigma } & =p_{M}\left(1\right)R_{\Sigma ,1}+p_{M}\left(2\right)\left(R_{\Sigma ,1}+R_{\Sigma ,2}\right)\\ & ={\bar{p}}_{M}\left(1\right)R_{\Sigma ,1}+{\bar{p}}_{M}\left(2\right)R_{\Sigma ,2}, \end{array}$$

with the notations ${\bar{p}}_{M}\left(1\right)=p_{M}\left(1\right)+p_{M}\left(2\right)$ and ${\bar{p}}_{M}\left(2\right)=p_{M}\left(2\right)$. The expected sum-rate ${\bar{R}}_{\Sigma }$ can be expressed as a function of $R_{F}$, P and $\left\{\Omega ,\Omega_{0}\right\}$:

(18) $$\begin{array}{cc} {\bar{R}}_{\Sigma } & =f_{\Sigma }\left(R_{F},P,\Omega ,\Omega_{0}\right)\\ & ={\bar{p}}_{M}\left(1\right)f_{1}\left(P,\Omega ,\Omega_{0}\right)+{\bar{p}}_{M}\left(2\right)f_{2}\left(P,\Omega ,\Omega_{0}\right). \end{array}$$

We note that increasing the compression output rate $R_{F}$ has conflicting effects on the expected sum-rate ${\bar{R}}_{\Sigma }$. On the one hand, the probability of timely reception of all fronthaul packets decreases with $R_{F}$ due to the increased number $N_{F}$ of packets in (8). On the other hand, once the packets have arrived at the cloud, a better sum-rate can be achieved with larger $R_{F}$, since the quantization noise signals have smaller powers.

The problem mentioned above can be stated as

(19a) $$\begin{array}{cc} \underset{R_{F},P,\Omega ,\Omega_{0}}{\mathrm{maximize}} & \;\;\;f_{\Sigma }\left(R_{F},P,\Omega ,\Omega_{0}\right) \end{array}$$

(19b) $$\begin{array}{cc} s.t.\;\;\; & R_{F}\geq g_{1}\left(\Omega ,\Omega_{0}\right), \end{array}$$

(19c) $$\begin{array}{cc} & 2R_{F}\geq g_{\mathrm{sum}}\left(\Omega ,\Omega_{0}\right), \end{array}$$

(19d) $$\begin{array}{cc} & \Omega ⪰0,\;\;\Omega_{0}⪰0, \end{array}$$

(19e) $$\begin{array}{cc} & P_{k,1}+P_{k,2}=P,\;\;k\in N_{U}, \end{array}$$

(19f) $$\begin{array}{cc} & P_{k,1}\geq 0,\;\;P_{k,2}\geq 0,\;\;k\in N_{U}. \end{array}$$

To tackle the problem (19), we first note that, if we fix the compression output rate variable $R_{F}$, the problem becomes a difference-of-convex (DC) problem as in [23]. Therefore, we can find an efficient solution by adopting the concave convex procedure (CCCP) approach (see, e.g., [24,25]). The detailed algorithm that tackles (19) with the CCCP approach is described in Algorithm 1, where we have defined the functions ${\tilde{f}}_{\Sigma }\left(R_{F},P,\Omega ,\Omega_{0},P^{\left(t\right)},\Omega^{\left(t\right)},\Omega_{0}^{\left(t\right)}\right)$, ${\tilde{g}}_{1}\left(\Omega ,\Omega_{0},\Omega^{\left(t\right)},\Omega_{0}^{\left(t\right)}\right)$ and ${\tilde{g}}_{\mathrm{sum}}\left(\Omega ,\Omega_{0},\Omega^{\left(t\right)},\Omega_{0}^{\left(t\right)}\right)$ as

$$\begin{array}{cc} {\tilde{f}}_{\Sigma }\left(R_{F},P,\Omega ,\Omega_{0},P^{\left(t\right)},\Omega^{\left(t\right)},\Omega_{0}^{\left(t\right)}\right)= & {\bar{p}}_{M}\left(1\right)\left(\begin{array}{c} \log_{2}\det\left(H\Sigma_{x}H^{H}+\Sigma_{z}+\Omega \right)\\ -\phi \left(\begin{array}{c} H{\bar{P}}_{2}H^{H}+\Sigma_{z}+\Omega ,\\ H{\bar{P}}_{2}^{\left(t\right)}H^{H}+\Sigma_{z}+\Omega^{\left(t\right)} \end{array}\right) \end{array}\right)\\ & +{\bar{p}}_{M}\left(2\right)\left(\begin{array}{c} \log_{2}\det\left(H{\bar{P}}_{2}H^{H}+\Sigma_{z}+\Omega_{0}\right)\\ -\phi \left(\Sigma_{z}+\Omega_{0},\Sigma_{z}+\Omega_{0}^{\left(t\right)}\right) \end{array}\right),\\ {\tilde{g}}_{1}\left(\Omega ,\Omega_{0},\Omega^{\left(t\right)},\Omega_{0}^{\left(t\right)}\right)= & \phi \left(\Sigma_{y}+\Omega ,\Sigma_{y}+\Omega^{\left(t\right)}\right)-\log_{2}\det\left(\Omega \right),\\ \mathrm{and}\;\;\;{\tilde{g}}_{\mathrm{sum}}\left(\Omega ,\Omega_{0},\Omega^{\left(t\right)},\Omega_{0}^{\left(t\right)}\right)= & \log_{2}\det\left(\Sigma_{y}\right)+\phi \left(A_{3}\Sigma_{y}A_{3}^{H}+\bar{\Omega },A_{3}\Sigma_{y}A_{3}^{H}+{\bar{\Omega }}^{\left(t\right)}\right)\\ & -\log_{2}\det\left(A_{4}\Sigma_{y}A_{4}^{H}+\mathrm{diag}\left(0_{n_{R}},\bar{\Omega }\right)\right)\\ & +2\phi \left(\Sigma_{y}+\Omega ,\Sigma_{y}+\Omega^{\left(t\right)}\right)-\log_{2}\det\left(A_{2}\Sigma_{y}A_{2}^{H}+I_{2}⊗\Omega \right), \end{array}$$

with the function $\phi \left(A,B\right)$ defined as

$$\begin{array}{cc} \phi \left(A,B\right) & =\log_{2}\det\left(B\right)+\frac{1}{\ln2}\mathrm{tr}\left(B^{-1}\left(A-B\right)\right). \end{array}$$

**Algorithm 1** CCCP algorithm for problem (19) for fixed $R_{F}$**1.** Initialize the variables $P^{\left(1\right)}$, $\Omega^{\left(1\right)},\Omega_{0}^{\left(1\right)}$ to arbitrary matrices that satisfy the constraints (19b), (19c) and (19d), and set t←1. **2.** Update the variables $P^{\left(t+1\right)},\Omega^{\left(t+1\right)},\Omega_{0}^{\left(t+1\right)}$ as a solution of the convex problem:
(20a)$$\begin{array}{cc} \underset{P,\Omega ,\Omega_{0}}{\mathrm{maximize}} & \;\;\;{\tilde{f}}_{\Sigma }\left(R_{F},P,\Omega ,\Omega_{0},P^{\left(t\right)},\Omega^{\left(t\right)},\Omega_{0}^{\left(t\right)}\right) \end{array}$$
(20b)$$\begin{array}{cc} s.t.\;\;\; & R_{F}\geq {\tilde{g}}_{1}\left(\Omega ,\Omega_{0},\Omega^{\left(t\right)},\Omega_{0}^{\left(t\right)}\right), \end{array}$$
(20c)$$\begin{array}{cc} & 2R_{F}\geq {\tilde{g}}_{\mathrm{sum}}\left(\Omega ,\Omega_{0},\Omega^{\left(t\right)},\Omega_{0}^{\left(t\right)}\right), \end{array}$$
(20d)$$\begin{array}{cc} & \Omega ⪰0,\;\;\Omega_{0}⪰0, \end{array}$$
(20e)$$\begin{array}{c} P_{1}+P_{2}=P, \end{array}$$
(20f)$$\begin{array}{c} P_{1}\geq 0,\;\;P_{2}\geq 0. \end{array}$$ **3.** Stop if a convergence criterion is satisfied. Otherwise, set t←t+1 and go back to Step 2.

We have discussed the optimization of the power allocation variables P and the quantization noise covariance matrices $\left\{\Omega ,\Omega_{0}\right\}$ for fixed compression output rate $R_{F}$. For the optimization of $R_{F}$, we propose to perform a 1-dimensional discrete search over $R_{F}\in R=\left\{\Delta_{R_{F}},2\Delta_{R_{F}},\ldots ,N_{F,\max}\Delta_{R_{F}}\right\}$ with $\Delta_{R_{F}}=B_{F}/L_{W}$ and $N_{F,\max}=T_{F}+1$. Here we have excluded the values $\tau \Delta_{R_{F}}$ with non-integer τ from the search space R. This does not cause a loss of optimality, since we can increase the compression output rate, hence improving the compression fidelity to $\left\lceil \tau \right\rceil \Delta_{R_{F}}$ without increasing the number $N_{F}$ of packets in (8) that needs to be delivered to the cloud.

### Optimization of Traditional Path-Diversity Scheme

In this subsection, we discuss the optimization of the traditional path-diversity (PD) scheme, in which the RRH repeats to send the same packet on the available two routes [11]. Accordingly, the RRH produces only a single quantized signal $\hat{y}=y+q$, where the quantization noise q is independent of y and distributed as q∼CN(0,Ω) under the assumption of standard Gaussian quantization codebooks. Denoting as $R_{F}$ the compression output rate for the quantized signal $\hat{y}$, the rate $R_{F}$ should satisfy the condition

(21) $$\begin{array}{cc} R_{F}\geq & g\left(\Omega \right)=I\left(y;\hat{y}\right)\\ & =\log_{2}\det\left(\Sigma_{y}+\Omega \right)-\log_{2}\det\left(\Omega \right). \end{array}$$

To evaluate the achievable sum-rate, we define the binary variable D∈{0,1}, which takes 1 if at least one packet arrives at the cloud, and 0 otherwise. The probability distribution of *D* can be written as

(22) $$\mathrm{Pr}\left[D=d\right]=\left\{\begin{array}{cc} P_{\emptyset }^{c}\left(T_{F}\right), & d=0\\ 1-P_{\emptyset }^{c}\left(T_{F}\right), & d=1 \end{array}\right..$$

If both packets sent on two routes are lost (i.e., D=0), the cloud cannot decode the signals sent by the UEs. If the cloud receives at least one packet (D=1), the cloud can perform decoding of the signals x based on the received quantized signal $\hat{y}$, and the achievable sum-rate can be written as

(23) $$\begin{array}{cc} R_{\Sigma } & =f_{\Sigma }\left(\Omega \right)=I\left(x;\hat{y}\right)\\ & =\log_{2}\det\left(H\Sigma_{x}H^{H}+\Sigma_{z}+\Omega \right)-\log_{2}\det\left(\Sigma_{z}+\Omega \right). \end{array}$$

The expected sum-rate ${\bar{R}}_{\Sigma }$ can be expressed as

(24) $$\begin{array}{cc} {\bar{R}}_{\Sigma } & =f_{\Sigma }\left(R_{F},\Omega \right)=\mathrm{Pr}\left[D=1\right]f_{\Sigma }\left(\Omega \right). \end{array}$$

The problem of maximizing the expected sum-rate ${\bar{R}}_{\Sigma }$ with the traditional PD scheme can hence be stated as

(25a) $$\begin{array}{cc} \underset{R_{F},\Omega }{\mathrm{maximize}} & \;\;\;f_{\Sigma }\left(R_{F},\Omega \right) \end{array}$$

(25b) $$\begin{array}{cc} s.t.\;\;\; & R_{F}\geq g\left(\Omega \right), \end{array}$$

(25c) $$\begin{array}{cc} & \Omega ⪰0. \end{array}$$

We can tackle the problem (25) in a similar approach to that proposed for addressing (19).

## 5. Numerical Results

In this section, we provide numerical results that validate the advantages of the proposed robust baseband compression technique based on MDC coding scheme. We consider a system bandwidth of 100 MHz and assume that each wireless frame consists of $L_{W}=5000$ channel uses. We also assume that each fronthaul packet has $B_{F}=6000$ bits (i.e., 750 bytes) which corresponds to a half of the maximum payload size per frame defined in Ethernet [10]. Denoting as $C_{F}$ the fronthaul capacity in bit/s, each fronthaul packet has the duration of $B_{F}/C_{F}$. If we define the maximum tolerable delay on fronthaul network as $T_{\max}$ s, the deadline $T_{F}$ in packet duration is given as $T_{F}=\left\lfloor T_{\max}/\left(B_{F}/C_{F}\right)\right\rfloor$. In the simulation, we set $T_{\max}=1$ ms. For simplicity, we assume that all paths have the same error probability $\epsilon_{F,l}=\epsilon_{F}$ for all l∈{1,2}. Regarding the channel statistics, we assume that the positions of the UEs and the RRH are uniformly distributed within a circular area of radius 100 m. The elements of the channel matrix $H_{k}$ are independent and identically distributed (i.i.d.) as $\mathrm{CN}(0,\rho_{k})$. Here the path-loss $\rho_{k}$ is modeled as $\rho_{k}=1/\left(1+{\left(d_{k}/d_{0}\right)}^{3}\right)$, where $d_{k}$ represents the distance between the RRH and UE *k*, and $d_{0}$ is the reference distance set to $d_{0}=30$ m. We set the noise covariance to $\Sigma_{z}=N_{0}I_{n_{R}}$, and the SNR is defined as $P/N_{0}$.

### 5.1. Fixed Compression Output Rate $R_{F}$

**Figure 5:**
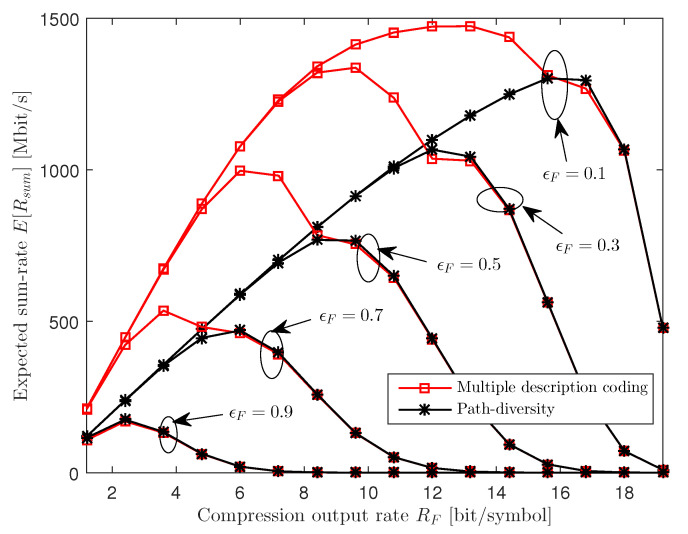
Expected sum-rate $E[R_{\mathrm{sum}}]$ versus the compression output rate $R_{F}$ for various values of $\epsilon_{F}\in \left\{0.1,0.3,0.5,0.7,0.9\right\}$ ($N_{U}=2$, $n_{R}=2$, $n_{U,k}=1$, $C_{F}=100$ Mbit/s and 25 dB signal-to-noise ratio (SNR)).

We first evaluate the expected sum-rate performance $E[R_{\mathrm{sum}}]$ when only the power allocation variables P and the quantization noise covariance matrices Ω are optimized according to Algorithm 1 for fixed compression output rate $R_{F}$. In Figure 5, we plot the expected sum-rate $E[R_{\mathrm{sum}}]$ versus the compression output rate $R_{F}$ for various values of path error probability $\epsilon_{F}$ with $N_{U}=2$, $n_{R}=2$, $n_{U,k}=1$, $C_{F}=100$ Mbit/s and 25 dB SNR. We observe that, for both the MDC and PD schemes, the optimal compression output rate $R_{F}$ increases as the fronthaul error probability $\epsilon_{F}$ decreases. This suggests that, with smaller $\epsilon_{F}$, the packet networks become more reliable and hence more packets can be reliably delivered to the cloud within the deadline. Furthermore, the figure shows that, with MDC, it is optimal to choose a lower compression output rate with respect to PD. This is because, as the fronthaul quality improves in terms of the error probability $\epsilon_{F}$, the PD scheme can only increase the sum-rate by increasing the quality, or the compression output rate $R_{F}$, of each individual description, since it cannot benefit from reception of both descriptions. In contrast, the MDC scheme can operate at a lower $R_{F}$, since the quality of the compressed signal is improved by reception of both descriptions. Receiving both descriptions tends to be more likely if the compression output rate is lower and hence the number of fronthaul packets per frame is reduced.

**Figure 6:**
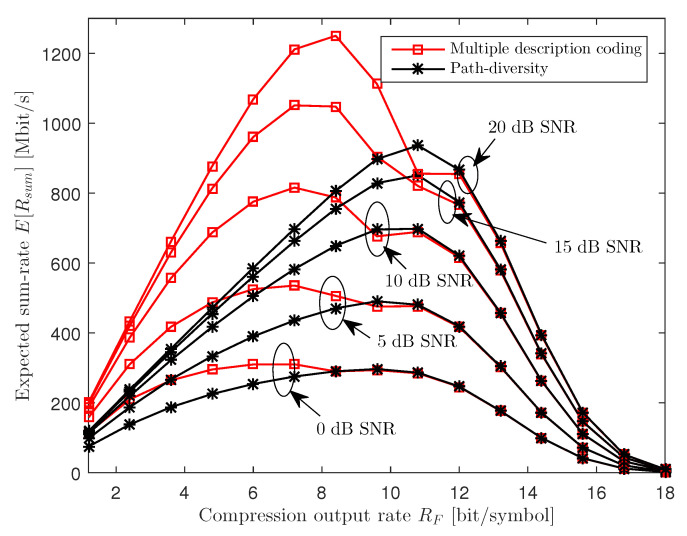
Expected sum-rate $E[R_{\mathrm{sum}}]$ versus the compression output rate $R_{F}$ for various SNR levels ($\epsilon_{F}=0.4$, $N_{U}=3$, $n_{R}=3$, $n_{U,k}=1$ and $C_{F}=100$ Mbit/s).

In Figure 6, we depict the expected sum-rate $E[R_{\mathrm{sum}}]$ versus the compression output rate $R_{F}$ for various SNR levels with $\epsilon_{F}=0.4$, $N_{U}=3$, $n_{R}=3$, $n_{U,k}=1$ and $C_{F}=100$ Mbit/s. The figure shows that, as the SNR increases, the optimal compression output rate $R_{F}$ slightly increases for both MDC and PD. This is because, while the SNR level does not affect the reliability of the packet fronthaul network, it is desirable for the RRH to report better descriptions of the uplink received signals to the cloud when the received signals carry more information on the UEs’ messages.

**Figure 7:**
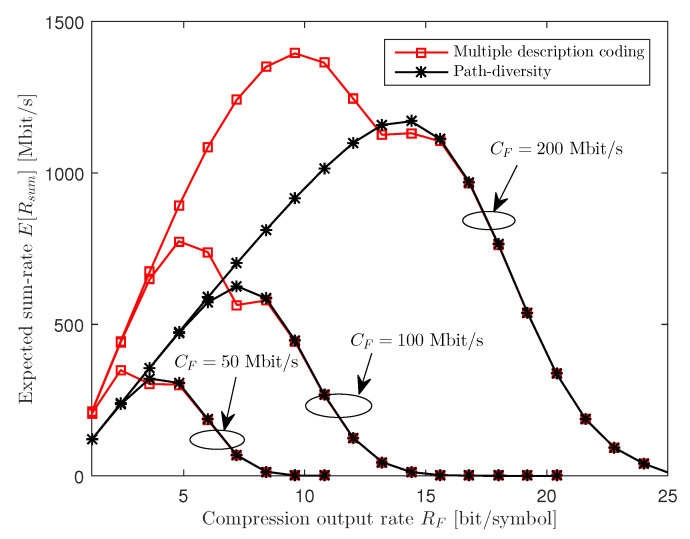
Expected sum-rate $E[R_{\mathrm{sum}}]$ versus the compression output rate $R_{F}$ for various fronthaul capacity $C_{F}$ ($\epsilon_{F}=0.6$, $N_{U}=2$, $n_{R}=2$, $n_{U,k}=1$ and 25 dB SNR).

Figure 7 plots the expected sum-rate $E[R_{\mathrm{sum}}]$ with respect to the compression output rate $R_{F}$ for various fronthaul capacity $C_{F}$ with $\epsilon_{F}=0.6$, $N_{U}=2$, $n_{R}=2$, $n_{U,k}=1$ and 25 dB SNR. Since more packets, and hence more bits, can be transferred to the cloud within the deadline $T_{F}$ with increased fronthaul capacity $C_{F}$, the optimal compression output rate $R_{F}$ grows with $C_{F}$ for both the MDC and PD schemes.

### 5.2. Optimized Compression Output Rate $R_{F}$

**Figure 8:**
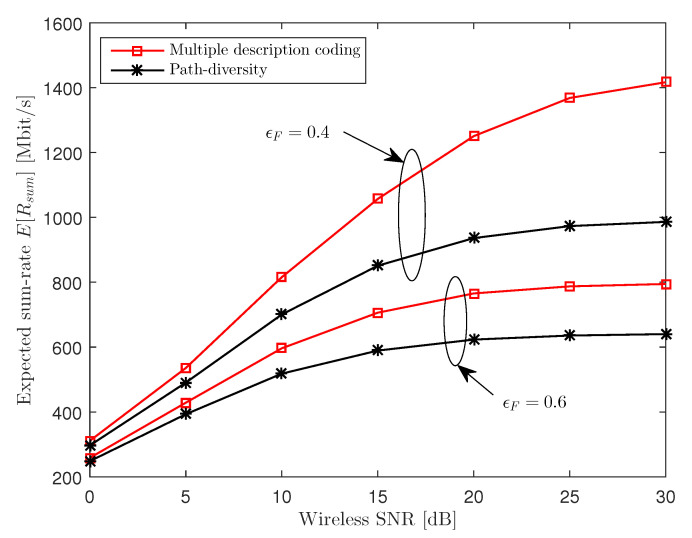
Expected sum-rate $E[R_{\mathrm{sum}}]$ versus the SNR ($N_{U}=3$, $n_{R}=3$, $n_{U,k}=1$, $\epsilon_{F}\in \left\{0.4,0.6\right\}$ and $C_{F}=100$ Mbit/s).

In this subsection, we present the expected sum-rate $E[R_{\mathrm{sum}}]$ achieved when the power allocation variables P, the quantization noise covariance matrices Ω and the compression output rate $R_{F}$ are jointly optimized as discussed in Section 4. In Figure 8, we plot the expected sum-rate $E[R_{\mathrm{sum}}]$ versus the SNR for $N_{U}=3$, $n_{R}=3$, $n_{U,k}=1$, $\epsilon_{F}\in \left\{0.4,0.6\right\}$ and $C_{F}=100$ Mbit/s. We observe from the figure that the MDC scheme shows a larger gain at a higher SNR level. This suggests that, as the SNR increases, the overall performance becomes limited by the quantization distortion which is smaller for the MDC scheme than for PD.

**Figure 9:**
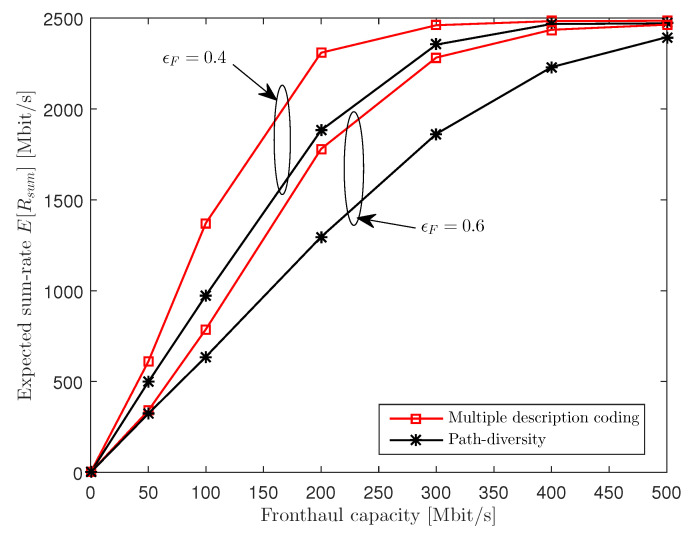
Expected sum-rate $E[R_{\mathrm{sum}}]$ versus the fronthaul capacity $C_{F}$ ($N_{U}=3$, $n_{R}=3$, $n_{U,k}=1$, $\epsilon_{F}\in \left\{0.4,0.6\right\}$ and 25 dB SNR).

In Figure 9, we plot the expected sum-rate $E[R_{\mathrm{sum}}]$ versus the fronthaul capacity $C_{F}$ for $N_{U}=3$, $n_{R}=3$, $n_{U,k}=1$, $\epsilon_{F}\in \left\{0.4,0.6\right\}$ and 25 dB SNR. The figure illustrates that the MDC scheme shows relevant gains over the PD scheme in the intermediate regime of $C_{F}$. This is because, when the fronthaul capacity $C_{F}$ is sufficiently large, the whole system has a performance bottleneck in the wireless uplink rather than in the fronthaul network, and the sum-rate converges to 0 as $C_{F}$ approaches 0.

## 6. Extension to General Numbers of RRHs and Fronthaul Paths

In this section, we briefly discuss the application of MDC to the case of general number $N_{R}$ of RRHs and $N_{P}$ fronthaul paths. Each RRH *i* sends $N_{P}$ descriptions ${\hat{y}}_{i,l}$, $l\in \{1,\ldots ,N_{P}\}$, one on each of the routes to the cloud, where ${\hat{y}}_{i,l}$ is a quantized version of the received signal $y_{i}$ defined as

(26) $$\begin{array}{c} {\hat{y}}_{i,l}=y_{i}+q_{i,l}. \end{array}$$

As in (5), under Gaussian quantization codebook, the quantization noise $q_{i,l}$ is independent of $y_{i}$ and is distributed as $q_{i,l}∼\mathrm{CN}\left(0,\Omega_{i}\right)$. With MDC, the cloud can recover the signal ${\hat{y}}_{i,l}$ if only the packets for the *l*th description ${\hat{y}}_{i,l}$ arrive at the cloud within the deadline. If a subset of descriptions from RRH *i* arrive in time, the cloud can obtain a better signal from RRH *i*, whose quality increases with the size of the subset. Generalizing (2)–(4), conditions relating the resulting quantization noise covariance matrices and the output compression rate $R_{F}$ can be found in [26].

We define as $M_{i}\in \left\{0,1,\ldots ,N_{P}\right\}$ the number of descriptions of RRH *i* that arrive at the cloud within the deadline $T_{F}$. The probability distribution $p_{M_{i}}\left(m\right)=\mathrm{Pr}\left[M_{i}=m\right]$ of $M_{i}$ is then given as

(27) $$\begin{array}{c} p_{M_{i}}\left(m\right)=\sum_{\left(c_{1},\ldots ,c_{N_{P}}\right)\in {\left\{0,1\right\}}^{N_{P}}}1\;\left(\sum_{l=1}^{N_{P}}c_{l}=m\right)\prod_{l=1}^{N_{P}}{\tilde{P}}_{l}\left(T_{F}\right), \end{array}$$

where 1(·) is an indicator function that outputs 1 if the input statement is true and 0 otherwise; and the probability ${\tilde{P}}_{l}\left(T_{F}\right)$ is defined as ${\tilde{P}}_{l}\left(T_{F}\right)=1\left(c_{l}=1\right)P_{l}^{c}\left(T_{F}\right)+1\left(c_{l}=0\right)\left(1-P_{l}^{c}\left(T_{F}\right)\right)$.

As discussed, with MDC, the quality of the information available at the cloud depends on the numbers of descriptions that arrive at the cloud. This means that there are ${\left(N_{P}+1\right)}^{N_{R}}$ distinct states depending on the current congestion level of the packet network. In principle, the broadcast coding can be applied in such a way that each UE *k* sends a superposition of ${\left(N_{P}+1\right)}^{N_{R}}$ layers. However, this approach does not scale well with respect to $N_{R}$, and it is not straightforward to rank all the ${\left(N_{P}+1\right)}^{N_{R}}$ states.

To adopt a broadcast coding strategy with a scalable complexity, a possible option is to fix the number of layers, denoted as *L*, as in, e.g., [27]. Accordingly, the transmit signal $x_{k}$ of each UE *k* is given by a superposition of *L* independent signals $x_{k,l}∼\mathrm{CN}\left(0,P_{k,l}I_{n_{U,k}}\right)$, l∈L={1,…,L}, i.e., $x_{k}=\sum_{l\in L}x_{k,l}$ with the power constraint $\sum_{l\in L}P_{k,l}=P$. We then partition the ${\left(N_{P}+1\right)}^{N_{R}}$ congestion states into *L* groups, denoted as $S_{1},\ldots ,S_{L}$, so that the layer-*l* signals ${\left\{x_{k,l}\right\}}_{k\in N_{U}}$ can be decoded by the cloud for all congestion states in $S_{j}$ with j≥l. Since we can evaluate the probability of all the states using (27), the expected sum-rate can be expressed as a function of the compression output rate, the power allocation variables and the quantization covariance matrices. Therefore, we can tackle the problem of jointly optimizing these variables in a similar approach to that proposed in Section 4. We leave the evaluation of the impacts of the numbers of RRHs $N_{R}$ and fronthaul paths $N_{P}$ to future work.

## 7. Conclusions

In this paper, we have studied the joint design of uplink radio and fronthaul packet transmission strategies for the uplink of C-RAN with a packet-based fronthaul network. To efficiently use multiple fronthaul paths that carry fronthaul packets from RRHs to cloud, we have proposed an MDC scheme that operates directly on the baseband signals. Since the signal quality available at the cloud depends on the current network congestion level, a broadcast coding strategy has been investigated with MDC in order to enable variable-rate transmission. The advantages of the proposed MDC scheme compared to the traditional PD technique have been validated through extensive numerical results. Among open problems, we mention the analysis in the presence of imperfect channel state information [28], the impact of joint decompression of the signals received from multiple RRHs at the cloud [3,23], and design of downlink transmission for C-RAN systems with packet-based fronthaul network.
